# Addictive drugs and plasticity of glutamatergic synapses on dopaminergic neurons: what have we learned from genetic mouse models?

**DOI:** 10.3389/fnmol.2012.00089

**Published:** 2012-08-31

**Authors:** Jan Rodriguez Parkitna, David Engblom

**Affiliations:** ^1^Department of Molecular Neuropharmacology, Institute of Pharmacology of the Polish Academy of SciencesKrakow, Poland; ^2^Department of Clinical and Experimental Medicine, Linköping UniversityLinköping, Sweden

**Keywords:** addiction, dopamine, genetically modified mice, glutamate, neural plasticity

## Abstract

Drug-induced changes in the functional properties of neurons in the mesolimbic dopaminergic system are attractive candidates for the molecular underpinnings of addiction. A central question in this context has been how drugs of abuse affect synaptic plasticity on dopaminergic cells in the ventral tegmental area. We now know that the intake of addictive drugs is accompanied by a complex sequence of alterations in the properties of excitatory synapses on dopaminergic neurons, mainly driven by signaling and redistribution of NMDA- and AMPA-receptors. It has, however, been unclear how these molecular changes are related to the behavioral effects of addictive drugs. Recently, new genetic tools have permitted researchers to perform genetic intervention with plasticity-related molecules selectively in dopaminergic cells and to subsequently study the behaviors of genetically modified mice. These studies have started to reveal how plasticity and drug-induced behavior are connected as well as what role plasticity in dopaminergic cells may have in general reward learning. The findings thus far show that there is not a one-to-one relation between plastic events and specific behaviors and that the early responses to drugs of abuse are to a large extent independent of the types of synaptic plasticity so far targeted. In contrast, plasticity in dopaminergic cells indeed is an important regulator of the persistence of behaviors driven by drug associations, making synaptic plasticity in dopaminergic cells an important field of study for understanding the mechanisms behind relapse.

## Introduction

The intake of drugs of abuse initiates progressive molecular changes in different parts of the brain. Whereas, some of these changes are associated with addiction, others relate to physiological or behavioral phenotypes that are not critically involved in the development of persistent drug-induced behaviors. However, distinguishing the changes associated with addiction from those merely correlated with drug intake has remained a challenge. Here, we focus on drug-induced synaptic strengthening of excitatory synapses on dopaminergic cells, a drug-induced change identified with electrophysiological techniques, and discuss how modern genetic intervention techniques have been used to elucidate the function of such plasticity. First, we will provide a brief summary of the genetic tools used, highlighting their pros and cons, and subsequently, we will discuss results from studies in which they have been used to assay the function of synaptic plasticity on dopaminergic cells, both in the context of cocaine addiction as well as in the setting of natural motivated behaviors.

## The genetic toolbox

Methods employed in the generation of genetically modified mice may be categorized based on the approach to introduce the mutation. The first approach is the replacement of a specific sequence in the genome by another, known as homologous recombination. This method involves transfection of embryonic stem cells with a DNA construct harboring a fragment that will replace the endogenous sequence flanked by homologous targeting sequences. The cells with recombined target sequence are then injected into mouse embryos in the blastocyst stage and transferred into a foster mother. Some of the offspring will be able to transmit the mutation, thus “founding” the mutated strain. This is the strategy employed in the generation of knockout (“KO”) animals. It also allows for the introduction of special sequences such as loxP sites to intronic regions flanking critical parts of a gene (a “floxed” gene) (Abremski et al., 1983; Thomas and Capecchi, 1987; Gu et al., 1994). Homologous recombination is also used to replace parts of a gene with another sequence (a so called “knockin”) such as a recombinase. Until recently, it was only possible to produce embryonic stem cells from specific mouse strains, such as 129 or FVB. Thus, many “KO” or “flox” lines have mixed strain background if they were not back-crossed over several generations. This may occasionally be a confounding factor, considering the differences in behavioral phenotypes of the 129 vs. C57 strains and, in particular, their different sensitivities to reinforcement and learning abilities (Crabbe et al., 2010).

**Figure 1:**
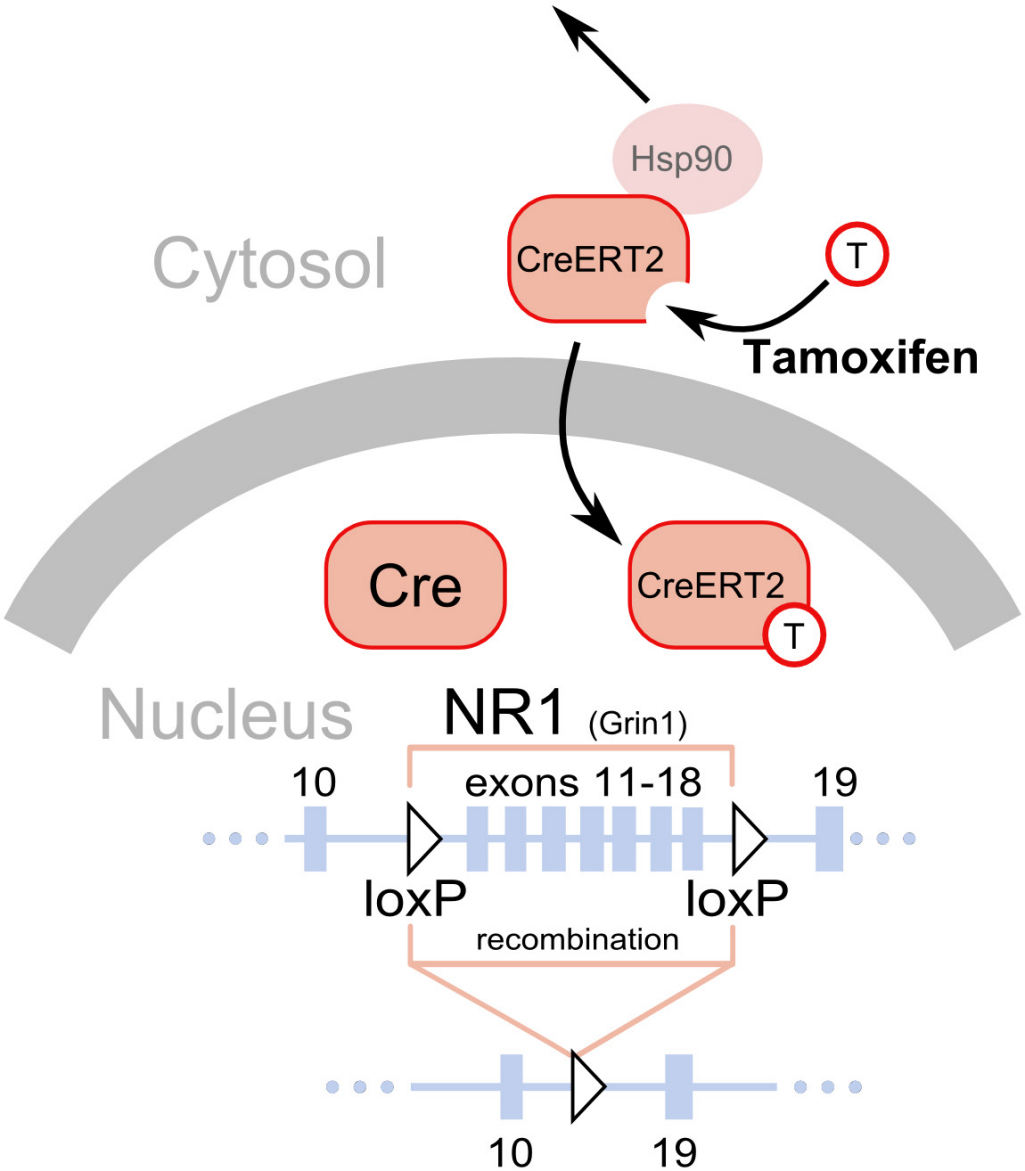
**Deletion of loxP flanked sequences by the Cre recombinase.** A fragment of the NR1 (*Grin1*) gene is shown as a line with solid black rectangles representing exons. The numbers above the rectangles correspond to exons. The two triangles represent the loxP sequences introduced in introns, placed in the same orientation and flanking exons 11–18 (Niewoehner et al., 2007). The Cre recombinase has a nuclear localization signal and is normally shuttled to the nucleus after translation, where it catalyzes a deletion of the gene fragment flanked by the loxP sites. Thus, gene inactivation will occur soon after the gene promoter driving Cre expression becomes active, typically around the 13th day of embryonic development (Parlato et al., 2006). The CreERT2 is a fusion protein of the Cre and a modified ligand binding domain of the estrogen receptor (ERT2). The modification prevents binding of endogenous estrogens but allows binding of tamoxifen, a synthetic steroid (represented by a circle with a “T”). Additionally, the presence of the ERT2 domain enables interaction with the mechanisms normally responsible for keeping the estrogen receptor in the cytosol, in particular interaction with Hsp90. Binding of tamoxifen to the ERT2 releases its interaction with the cytosolic proteins and permits shuttling to the nucleus, where it catalyzes the deletion of the gene fragment flanked by loxP sequences.

**Figure 2:**
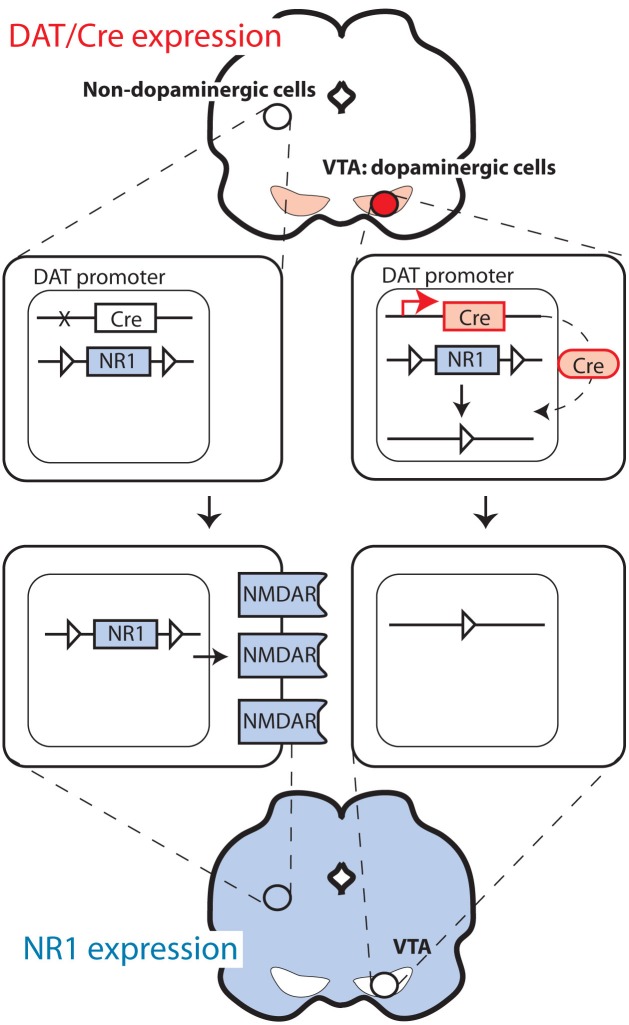
**Deletion of NMDA receptors selectively in dopaminergic cells**.

A second approach involves random insertion of a new DNA fragment (i.e., a transgene) in the genome. This is achieved by injecting a DNA construct into the prozygote (pronuclear injection) upon which a fraction of the offspring born from injected embryos will carry the transgene randomly, but stably, as it has been incorporated into their genome. This method has numerous applications (Branda and Dymecki, 2004; Dymecki and Kim, 2007). It is frequently used to introduce the Cre recombinase, an enzyme derived from bacteriophage P1, which belongs to the family of topoisomerases and has the ability to cut and ligate DNA strands (Abremski et al., 1983). Cre recognizes specific sequences, the loxP sites, which are not normally present in the murine genome. When a mouse with a recombinase transgene under the control of a cell-type specific promoter is crossed with an animal that contains a gene containing loxP sequences (created by homologous recombination), a deletion in the target gene will occur only in Cre-expressing cells (see Figures 1, 2). For mouse lines generated by pronuclear injection, the milieu surrounding the site of integration may affect both the level of transgene transcription as well as the cell-type specificity of expression. This may be circumvented in most cases by introducing large transgenes (over 100,000 DNA bases) based on bacterial artificial chromosomes (BACs) in which long flanking sequences buffer the positional effects (Yang et al., 1997).

For studies on the functional role of plasticity in dopaminergic cells, it is of pivotal importance to be able to delete genes selectively in dopaminergic cells. This is typically performed with mouse lines expressing the Cre recombinase under the control of the promoter of the dopamine transporter (DAT), also called *Slc6a3*. The strategies employed differ considerably. The knock-in approach in which the Cre sequence is replacing one DAT allele was used to generate one of the frequently used strains (Zhuang et al., 2005; Zweifel et al., 2008). This approach is advantageous in that it typically offers high fidelity in the pattern of recombinase expression (i.e., only in dopaminergic cells). However, removing one allele of a gene is sometimes sufficient to produce a change in behavior or physiology, as indeed was reported in the case of DAT (Jones et al., 1998). In a second line, this problem was avoided by introducing the Cre recombinase after the DAT encoding sequence, separated by an IRES sequence to allow translation of both the DAT and the Cre sequence (Backman et al., 2006). In another mouse line, Cre was expressed from a large transgene (Parlato et al., 2006), as well as also using the CreERT2 modification (Rieker et al., 2011), which prevents recombination until animals are treated with the synthetic steroid tamoxifen (Feil et al., 1996; Engblom et al., 2008) (Figure 1). Finally, Cre has also been targeted to regions with dopaminergic cells using stereotaxic injection of viral vectors (Zweifel et al., 2008), thus limiting the recombination to cells infected by the virus. This method allows targeting of very precise brain areas with the mutation (i.e., only VTA instead of all DAT-expressing cells in the body). However, this approach is often limited by problems with recombination efficiency and limited selectivity (i.e., all neuron types instead of only DAT-expressing).

The reported efficiency of recombination by the DAT-driven Cre recombinases was excellent, affecting essentially all dopaminergic cells (Engblom et al., 2008; Zweifel et al., 2008; Luo et al., 2010; Rieker et al., 2011). Nevertheless, it was also reported that the offspring of an animal carrying the transgene and one floxed allele of the target gene would frequently show complete deletion of the allele (Δ/flox; so-called “germline” recombination). This could be due to transient activity of the DAT promoter during very early development or gamete formation. The frequency of such events was reported to be highest in mice where the Cre replaces one of the DAT alleles. To prevent the germ-line deletion from confounding the behavioral analyses, additional genotyping is performed to remove affected mice or control groups including heterozygous animals (i.e., Δ/flox without the Cre transgene) are added to the experimental design. Moreover, scattered Cre-mediated recombination is sometimes observed in brain areas not expressing DAT, as even transient activity of the transgene may be sufficient to drive the mutation. This type of activity has accumulating effects with age, often being more pronounced in older animals.

In plasticity studies, dopamine cell-specific deletions have mostly targeted NMDA receptors. These studies are based on partial deletions of the gene encoding the essential NR1 receptor subunit (*Grin1*) using modified variants of the gene with loxP sequences introduced in introns after exons 10 and 18 (Niewoehner et al., 2007), 10 and 23 (Zweifel et al., 2008), or 11 and 21 (Tsien et al., 1996). Deletion of molecules that are central for neural signaling, such as NMDARs, may cause compensatory changes in the activity of targeted neurons due to the recombination occurring early in the development of the neurons. In the case of the Cre/loxP-mediated ablations of NR1 in dopaminergic cells, which has already occurred in the second week of embryonic development, the mutation caused an increase in spontaneous activity of dopaminergic cells measured in slice preparations (Engblom et al., 2008; Zweifel et al., 2008). This problem could be circumvented by the use of the inducible CreERT2 variant previously described. However, as with all approaches described in this section, this method is not without pitfalls. First, so-called “leakiness,” a level of recombination occurring without tamoxifen treatment, is a frequent problem. Second, tamoxifen treatment requires multiple i.p. injections over several days and is stressful to the animal, possibly causing persistent changes in behavior (Vogt et al., 2008).

In conclusion, although genetic intervention is an elegant way to test a gene's association with a specific phenotype, no method is without caveats. The discrete differences between superficially similar approaches should not be discarded as a technical aspect irrelevant to the conclusions. Inference of associations between genes and phenotypes is not possible without very extensive and sometimes impractical control experiments. In fact, it is simpler to demonstrate that a gene is not essential to a specific phenotype. Unfortunately, this is often avoided or unreported.

## Synaptic plasticity in dopaminergic cells

**Figure 3:**
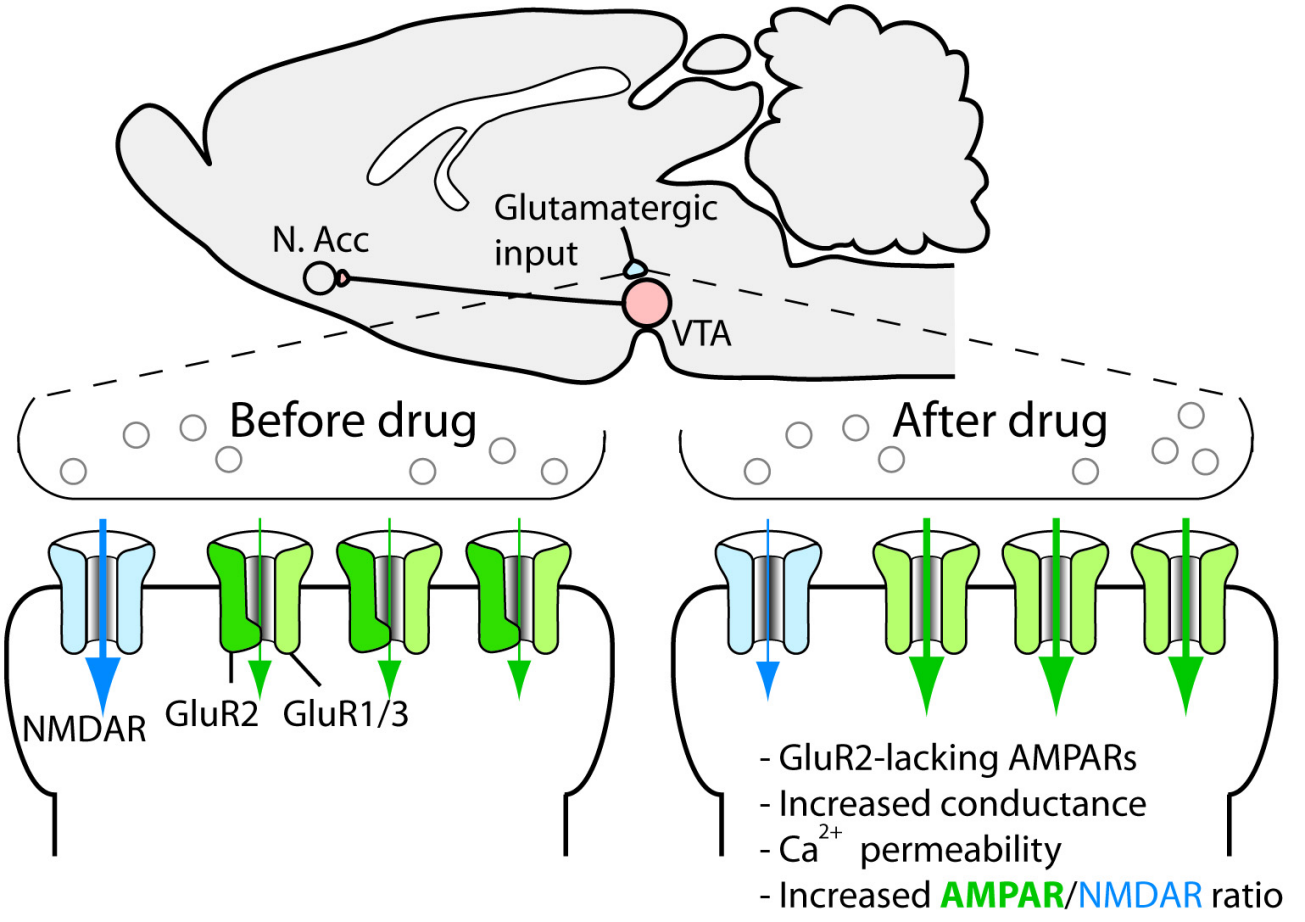
**Drug-induced synaptic strengthening of excitatory synapses on dopaminergic neurons**.

**Figure 4:**
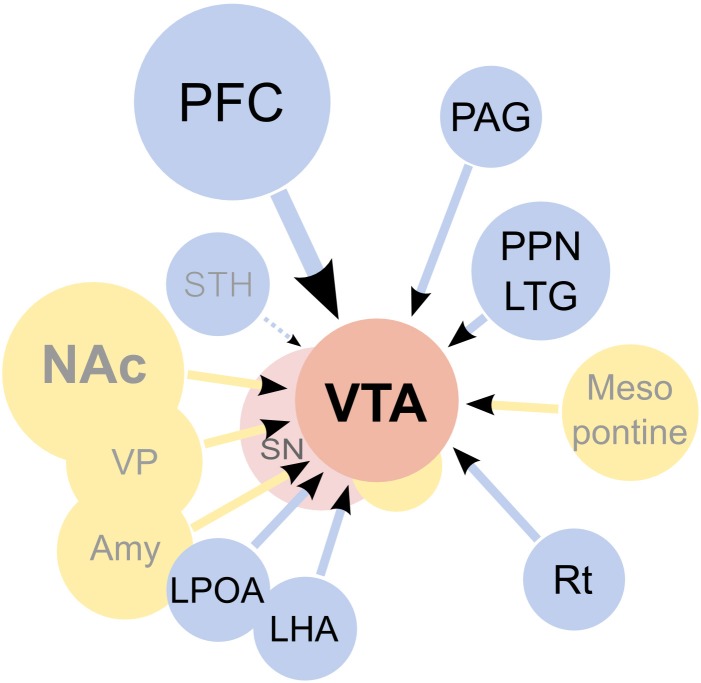
**Afferents to the ventral tegmental area (VTA).** On the diagram, origins of the main excitatory inputs are shown as blue circles, origins of the main inhibitory inputs as yellow circles, and the VTA and substantia nigra (SN) as red circles. Excitatory inputs originate from the prefrontal cortex (PFC), periaqueductal grey (PAG), the pedunculopontine nucleus (PPN), the laterodorsal tegmentum (LTG), lateral hypothalamus (LHA), lateral preoptic area (LPOA) and the reticular formation (Rt). The subthalamic nucleus (STH) was reported to send projections to the SN but not the VTA. It should be noted that the excitatory projections are forming synapses not only on dopaminergic cells, but on other types of neurons present in the VTA as well. GABAergic signaling regulates the activity of the VTA, both through local inhibitory neurons as well as afferents coming from the nucleus accumbens septi (NAc), ventral pallidum (VP), globus pallidus (GP), amygdaloid nuclei (Amy) and the mesopontine rostromedial tegmental nucleus. Additionally, a fraction of the afferents from the PPN was found to be GABAergic. The diagram does not show all inputs to the VTA, notably omitting serotonergic and cholinergic afferents and also inputs to non-dopaminergic neurons. For an excellent review of the architecture of the mesolimbic system please see Sesack and Grace (2010).

Synaptic plasticity, such as, long-term potentiation and long-term depression has received much attention since they may constitute cellular substrates of learning and memory (Malenka and Bear, 2004). Because aberrant reward learning has been proposed to be a key feature of drug addiction, drug-induced synaptic plasticity is a strong candidate for being critical for the development and persistence of addiction and other related behavioral responses to drugs of abuse (Everitt et al., 2001; Hyman et al., 2006; Luscher and Malenka, 2011). Given the pivotal role of dopaminergic cells in the rewarding actions of addictive drugs, these cells were a natural starting point in the search for drug-induced synaptic alterations. In a landmark study, Ungless et al. (2001) showed that a single injection of cocaine leads to a strengthening of excitatory synapses on dopaminergic cells of the ventral tegmental area, measured as an increase in the AMPA/NMDAR ratio in slices from the midbrain of mice (Figure 3). Later on, it was shown that all major addictive drugs can induce the same adaptation and that it is also induced by stress and reward-predicting cues (Saal et al., 2003; Stuber et al., 2008). The synaptic strengthening lasts for approximately 5 days after a single passive injection of cocaine (Ungless et al., 2001) and at least 3 months after a period of cocaine self-administration (Chen et al., 2008). Given the relatively limited life span of the rat, this is a very persistent change and is therefore interesting from a clinical perspective. A schematic representation of main excitatory and inhibitory inputs to the VTA is shown in Figure 4.

On the molecular level, it seems that everything that leads to increased dopamine levels in the VTA triggers the synaptic strengthening. Such increases are due to the dendritic release of dopamine from the dopaminergic cells and can be triggered by an increased firing rate of the dopaminergic cells, which is sufficient for the synaptic plasticity to occur, as shown by an optogenetic approach (Brown et al., 2010), or through the local release of dopamine due to interference with catecholamine transporters in the VTA (Argilli et al., 2008). Thus, we know that cocaine administered to midbrain slices induces synaptic strengthening and that administration of a D1/D5 receptor antagonist, as well as the deletion of the D5 receptor, blocks the strengthening (Argilli et al., 2008). Because midbrain dopaminergic neurons express D5 but not D1 receptors (Khan et al., 2000), the dopamine action is most likely mediated by D5 receptors on dopamine neurons, although a role for D1 receptors on afferents contacting the dopaminergic cells cannot be ruled out. In any case, NMDA receptors are necessary for the plasticity. Because deletion of NMDA receptors selectively on dopaminergic cells in adult mice is sufficient to block the strengthening (Engblom et al., 2008; Zweifel et al., 2008), we know that this is due to the disruption of NMDAR signaling on the dopaminergic cells themselves and that it is unlikely that the loss of plasticity is explained by adaptations during development. Further, the AMPA receptor subunit GluR1, located on the dopaminergic neurons, is necessary for the plasticity (Dong et al., 2004; Engblom et al., 2008). Careful studies of the induction mechanism strongly indicate that drugs trigger the release of dopamine, indirectly leading to NMDAR activation on dopaminergic cells. This in turn leads to the redistribution of AMPA receptors so that receptors containing the subunit GluR2 are exchanged for receptors that do not (e.g., GluR1 homotetramers) (Bellone and Luscher, 2006) (Figure 3). These receptors have a higher conductance and are permeable to calcium, and their incorporation leads to an increased responsiveness and also changes the rules for further strengthening at the synapse (Mameli et al., 2011). The AMPA receptor redistribution is accompanied by a reduction in functional NMDA receptors at the synapse (Mameli et al., 2011). If drug use is discontinued, an mGluR1-dependent mechanism resets the synapse to the original setup after around 1 week (Mameli et al., 2007). In addition to changing the receptive properties of the dopaminergic cell, this plasticity triggers plastic events in the nucleus accumbens (Mameli et al., 2009). The role of drug-induced synaptic adaptations in this structure has been reviewed elsewhere (Thomas et al., 2008; Wolf, 2010; Luscher and Malenka, 2011).

To describe synaptic strengthening at these synapses as a unified phenomenon is, of course, somewhat simplistic. The dopaminergic cells in the VTA receive input from many different structures (Figure 4) and have different projections. Thus far, very little is known about how connectivity affects plasticity in these cells, but dopaminergic neurons projecting to the cortex have been shown to be molecularly distinct from neurons projecting to the nucleus accumbens and are known to react with synaptic strengthening in response to aversive rather than rewarding stimuli (Lammel et al., 2011). Complicating matters even further, the synaptic strengthening described above is not the only form of drug-induced synaptic plasticity in dopaminergic cells. Of particular interest for the interpretation of functional studies addressing the role of the synaptic strengthening is the form of plasticity called LTP-GABA (Nugent et al., 2007). This plasticity is NMDAR-dependent and increases the release of GABA from terminals contacting the dopaminergic cells. Interestingly, morphine, cocaine, and nicotine inhibit LTP-GABA (Nugent et al., 2007). A postsynaptic form of GABA plasticity, leading to weaker inhibitory transmission, has also been described after repeated cocaine exposure (Liu et al., 2005; Pu et al., 2006). Although, much less is known about these forms of inhibitory plasticity, it is important to keep them in mind when interpreting studies that intervene in the functioning of plasticity-related molecules in dopaminergic cells because, to some extent, they share mechanisms.

## Functional studies using genetic tools

As previously noted, a lot of drug-induced molecular changes have no impact on behavior, and because addiction is a behavioral disorder, it is of pivotal importance to understand how different forms of synaptic plasticity contribute to behavior. However, this task is not trivial, and the different approaches used all have their weaknesses. One source of information is classical pharmacological studies in which NMDA or AMPA receptor antagonists are injected into the VTA. These studies indicate that NMDARs and AMPA receptors in the VTA are essential for both cocaine-induced behavioral sensitization (Kalivas and Alesdatter, 1993) and conditioned place preference (Harris and Aston-Jones, 2003). However, the problem with these studies is that they also target non-dopaminergic cells in the VTA and that the effects observed are not necessarily due to inhibited plasticity as they could be due to the disruption of steady state signaling. Thus, the results could imply that NMDAR signaling in response to the drug is necessary for triggering some type of adaptation that is essential for sensitization, but this change could be something completely different from synaptic strengthening on dopaminergic neurons. In this context, an important technical addition was the use of rats with viral mediated overexpression of GluR1 in the VTA (Carlezon et al., 1997). These rats should have synapses that are inherently strengthened. Indeed, they showed an increase in the locomotor activity in response to an acute injection of morphine, indicating that they were in a pre-sensitized state. Additionally, these animals displayed a potentiated CPP to morphine (Carlezon et al., 1997). Recently, it was also shown that such rats show increased motivation for self-administration of cocaine (Choi et al., 2011). The main problem with this type of study is that the forced overexpression of GluR1 is not physiological. To an extent, this has been solved by loss-of-function approaches using mice lacking GluR1. In these mice, the basal properties of the excitatory transmission on dopaminergic cells seem to be relatively unaltered—most likely due to the function of GluR3 compensating for the lack of GluR1. However, the synaptic strengthening is blocked in GluR1 KO mice (Dong et al., 2004), showing that GluR3 cannot compensate for GluR1 in this aspect. Interestingly, the GluR1 KO mice formed a perfectly normal sensitization to cocaine, whereas conditioned place preference was affected in one study (Dong et al., 2004) but not in others (Mead et al., 2005; Engblom et al., 2008). The major limitation of this approach is that it is impossible to know if an effect on behavior is due to the lack of GluR1 in dopaminergic cells or elsewhere. This is not only a theoretical problem because *a priori*, it would seem quite likely that GluR1-mediated plasticity in the nucleus accumbens, the amygdala or the hippocampus would be involved in reward-learning-related behaviors. Recent studies using Cre/loxP methodology have added some missing pieces to this puzzle, although the results are not always easy to fit into a coherent picture. Importantly, some of these studies also looked beyond the early drug-induced behaviors using relapse models. In one study (Engblom et al., 2008), mice with deletions of GluR1 (GluR1-DATCre), GluR2 (GluR2-DATCre), or NMDARs (NR1-DATCre) specific to dopamine neurons were used. The fact that the deletions are selective to dopaminergic cells is important since the aim was to investigate the role of plasticity in these specific cells but also since mice lacking NMDARs in the entire body die within the first day after birth (Forrest et al., 1994). As expected, cocaine-induced strengthening of excitatory synapses on dopaminergic cells was blocked in mice lacking GluR1 or NMDARs on dopaminergic cells, whereas, this effect was intact in mice lacking GluR2. Intriguingly, mice lacking GluR1 or NMDARs showed perfectly normal locomotor sensitization and CPP, indicating that the synaptic strengthening is not important for these early drug effects (Engblom et al., 2008). In contrast, mice lacking GluR1 in dopaminergic cells showed a blocked extinction of the CPP, and mice lacking NMDARs in dopaminergic cells showed a normal extinction but a blocked drug-induced reinstatement of the CPP. In addition, when NMDARs were deleted in dopaminergic cells of adult mice, to avoid the synaptic scaling induced by early removal of NMDARs, an identical block of reinstatement was observed. The inducible NMDAR mouse line (NR1-DATCreERT2) was later also used in a self-administration paradigm, the results of which were compatible with a role of NMDARs in dopaminergic cells in relapse (Mameli et al., 2009). In this case, the mutant mice showed normal levels of cocaine self-administration and normal extinction behaviors but reduced cue-induced reinstatement of cocaine-seeking. Thus, although there are also conflicting results (Luo et al., 2010), both of these studies are compatible with the view that NMDAR-dependent synaptic plasticity on dopaminergic cells is important for the persistence of drug seeking rather than the early responses to cocaine. Intriguingly, in another study, Zweifel et al. found a blockade of cocaine CPP in mice with deletion of NMDARs in dopaminergic cells (NR1-DATCre) and in mice with a local deletion of NMDARs in the VTA using a viral approach (Zweifel et al., 2008). The latter could be due to an effect on non-dopaminergic cells because NMDARs on non-dopaminergic cells in the VTA have been shown to regulate other cocaine-induced responses (i.e., locomotor sensitization) (Luo et al., 2010), but the blocked CPP in the NR1-DATCre mice at first glance contrasts sharply with the unaffected CPP observed by Engblom et al. and another study using different mouse lines (Luo et al., 2010). The differing results might reflect the fact that Zweifel et al. used mice with knockin-based Cre expression, thus destroying one of the DAT loci, and with heterozygous global deletion of NR1, both of which are known to affect behavior. Another possible reason is that in the CPP procedure used by Zweifel et al., every conditioning is followed by a drug-free test, mimicking an extinction session and making the subsequent conditioning quite similar to a reinstatement session. Thus, the CPP protocol used by Zweifel et al. actually has similarities to the reinstatement protocol used by Engblom et al., possibly indicating a reason for the deficiency observed in these two tests. Thus, it is quite clear from these studies that NMDARs in dopaminergic cells are not a universal requirement for cocaine CPP, but the findings of Zweifel et al. together with data on natural reward learning (discussed later) and nicotine CPP (Wang et al., 2010) indicate that it may be important for some associations under specific conditions.

**Figure 5:**
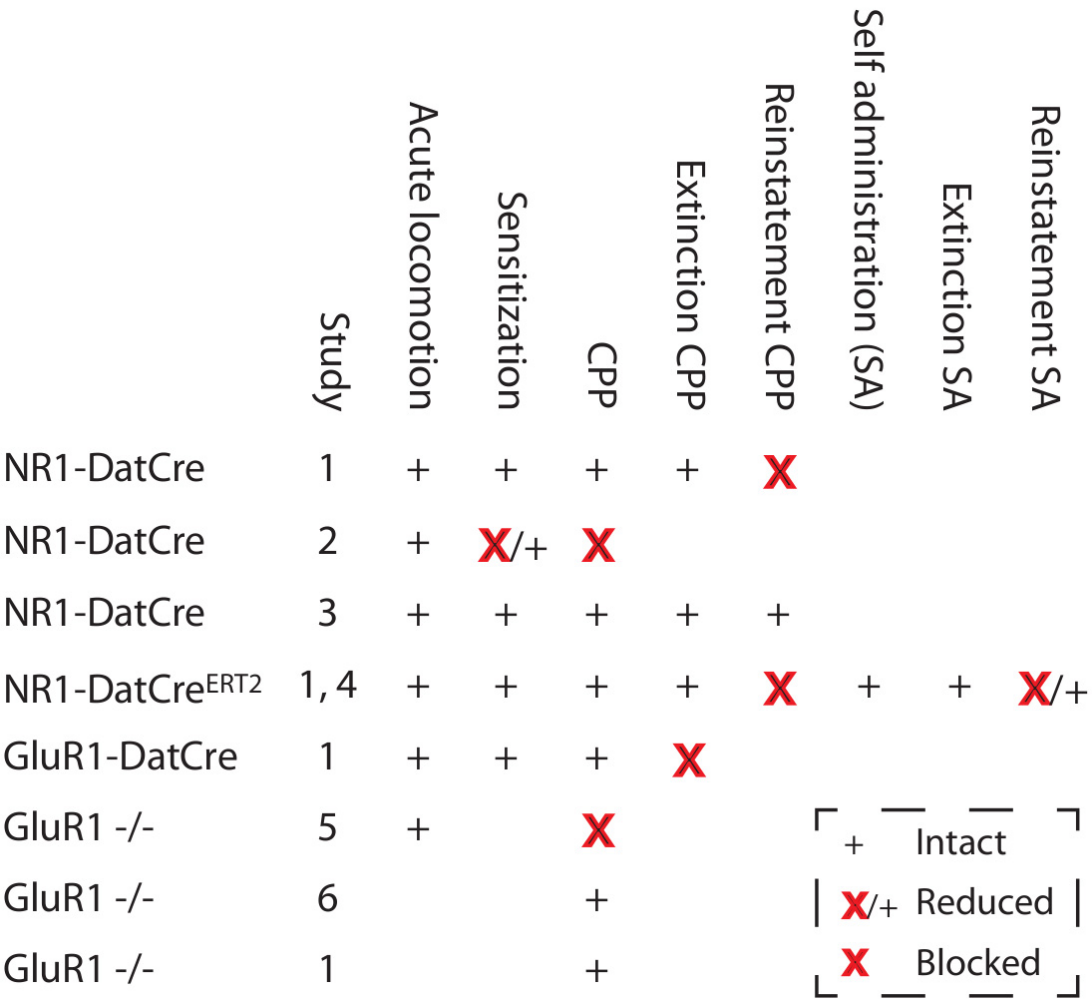
**Changes in cocaine-related behaviors in mice with deletions of NMDARs or GluR1.** Studies: 1, Engblom et al. (2008); 2, Zweifel et al. (2008); 3, Luo et al. (2010); 4, Mameli et al. (2009); 5, Dong et al. (2004); and 6, Mead et al. (2005). SA, Self administration.

Collectively (summarized in Figure 5), the most solid conclusion from these studies seems to be that for cocaine, NMDAR-dependent signaling in dopaminergic cells is important in reinstatement of both CPP and self-administration, indicating a possible role in relapse. However, we know very little about the mechanisms behind this involvement. For example, we do not know at which stage they are required. Along this line, we also do not know if the blocked reinstatement has anything to do with the fact that drug-induced synaptic strengthening is blocked. It could be that impaired LTP-GABA explains the lack of reinstatement or even that NMDAR signaling is required only during the reinstatement procedure and has a role unrelated to any plasticity. Moreover, as will be discussed later on, the mice lacking NMDARs in dopaminergic cells have also been used as a model to study a lack of burst firing, another potentially important phenomenon in the context of reinstatement. Unfortunately, because the GluR1-DATCre mice showed a blocked extinction, it was not possible to determine if they reinstate, which would have provided strong support for the idea that deficiencies in synaptic strengthening in dopaminergic cells are responsible for relapse. Thus, we cannot, at the present stage, prove any one-to-one correlation between synaptic strengthening and a behavioral phenotype, which may not be surprising given how difficult it has been to determine the relation of other even more well-characterized types of plasticity. Nevertheless, the data clearly point to a role for synaptic strengthening in the persistence of cocaine seeking, even if the exact mechanism remains partly unclear.

## The role of NMDARs on dopaminergic cells in motivated behaviors

It was observed that inactivation of NMDARs diminished burst (phasic) firing of DA neurons, without notably altering tonic activity (Zweifel et al., 2009; Wang et al., 2011). The scaling of excitatory synapses on DA neurons in mutant mice had no significant effect on dopamine tissue or extracellular levels (Engblom et al., 2008; Zweifel et al., 2008, 2009, 2011; Mameli et al., 2009). Hence, the mutant mice are an excellent model to distinguish the roles of tonic and burst activity in learning and motivated behaviors. Indeed, loss of NMDARs on dopamine neurons did not influence several behaviors that are known to depend on tonic dopamine signaling. NMDAR loss did not alter novelty-induced locomotor activity and recognition of a novel object, food consumption when chow is available *ad libitum*, social behaviors or prepulse inhibition (Zweifel et al., 2009). Mutant mice had normal learning abilities, as indicated by a normal latency to find a hidden platform in the Morris water maze and an intact ability to navigate in a T-maze task (Zweifel et al., 2009). Strikingly, loss of NR1 on dopaminergic cells did not affect acquisition of the conditioned approach during Pavlovian training (Parker et al., 2010), a behavior that was shown to involve phasic activity of dopamine cells and NMDAR-dependent synaptic-plasticity in the VTA (Stuber et al., 2008). Despite the lack of phasic activity mutant mice responded to the presentation of a cue previously signaling delivery of a reward, and presentation of the cue elicited dopamine release similar to that observed in control mice.

Conversely, the lack of burst-firing of dopamine neurons in mutant mice impaired their performance in several tasks dependent on salient cues or contexts, which included the cued water maze and T-maze tasks as well as fear-potentiated acoustic startle (Zweifel et al., 2009). They were also slower to learn the instrumental task in food self-administration but showed similar motivation to obtain food as assessed by the progressive ratio test, where the number of instrumental responses necessary to obtain a food reward increases with each reward administered (Zweifel et al., 2009). Nevertheless, in other mouse strains with an equivalent ablation of NMDAR in dopamine cells no impairment in learning of an instrumental task rewarded with cocaine (Mameli et al., 2009) or food (Wang et al., 2011) was reported. To some extent these discrepancies could be explained by a deficit of habit learning observed in mutant mice (Wang et al., 2011), which could affect their performance in tests with large numbers of repeated trials. Furthermore, differences in sensitivity to food vs. drug rewards were also observed in other behavioral tests. Mutant mice were found to have attenuated food CPP (Zweifel et al., 2009), even though most studies show that they develop a normal cocaine CPP (Engblom et al., 2008; Luo et al., 2010). Finally, the loss of NMDARs caused increased susceptibility to prolonged stress effects, as indicated by a persistently increased acoustic startle response and more anxiety-like behavior in the elevated plus maze test after aversive conditioning in mutant animals compared to controls (Zweifel et al., 2011). This phenotype could be prevented by injection of a viral vector that restored NR1 expression in dopamine cells, thus suggesting that impaired burst activity of VTA neurons may be involved in the development of anxiety disorders.

## Conclusion

Reports on behavioral phenotypes of mice with selective NR1 ablation in dopamine cells show selective roles of NMDA receptor plasticity and burst firing activity in reward learning. Many behaviors previously presumed to be associated with NMDA receptor-dependent long-term potentiation in dopamine neurons, such as, drug-conditioned place preference, psychomotor sensitization, or drug self-administration, are normal or mildly altered in mutant mice. Conversely, the loss of functional NMDA receptors prevented reinstatement of cocaine-conditioned place preference, attenuated reinstatement of cocaine self-administration and also generally impaired behaviors dependent on salient cues or contexts. Thus, despite differences in reported phenotypes, a possible conclusion is that NMDA receptors on dopamine cells are involved in the recall of previously learned behaviors in response to salient stimuli.

### Conflict of interest statement

The authors declare that the research was conducted in the absence of any commercial or financial relationships that could be construed as a potential conflict of interest.
